# On the Selection of a Climate for Patients with Pulmonary Tuberculosis

**Published:** 1888-10

**Authors:** Frederick I. Knight

**Affiliations:** Boston, Mass.; Assistant Professor, Harvard University


### ON THE SELECTION OF A CLIMATE FOR PATIENTS WITH PULMONARY TUBERCULOSIS.*

BY FREDERICK I. KNIGHT, M. D., ASSISTANT PROFESSOR, HARVARD UNIVERSITY.

### PREFACE.

I have often been asked to give the profession some brief practical hints on the choice of a climate for tuberculous patients. I am induced to comply with this request, although there is still a great diversity of opinion among those conversant with the sub-

*Read before the Boston Society for Medical Observation, March 5, 1888. *Boston Medical and Surgical Journal.*

ject, because there seems to be so little attempt even at any guidance of patients in this important matter.

I shall make no endeavor to review the constituents of climate, or to philosophize on their mode of action. I shall briefly present typical cases of pulmonary tuberculosis of different kinds, and point out how I should determine whether the patient should be sent away from home at all, and if sent, to what region of our own country.

I wish it to be distinctly understood that I am giving simply my own convictions on this important subject, based upon twenty years' experience, and that I do not claim that the principles which guide me are by any means universally accepted by climatologists. If I designate my special places for residence, it is because they offer some accommodations and are well known, but of course it will be understood that there are scores of similarly situated regions which would answer just as well as far as climatic conditions are concerned.

### SHALL PATIENTS THREATENED WITH, OR ALREADY HAVING, PULMONARY TUBERCULOSIS BE ADVISED TO CHANGE CLIMATE?

This is a serious question, not to be settled off-hand, and one entitled to much more consideration before being answered than is usually accorded it. Not only is serious inconvenience and great expense often unnecessarily caused a patient and his family by hasty advice in this regard, but his condition is not infrequently made worse by it. This may happen in cases in which rest at home would have been better than any change, or in which the destination has been improperly specified, or perhaps not specified at all. In every case the condition of the individual patient must be carefully considered, whether any climate change could possibly improve it, and if so, what kind of a change would be most likely to effect it.

Other considerations, however, besides the disease of the patient, have an important bearing upon the question of change. Can the patient afford it? The physician should satisfy himself on this point, if possible, before mentioning its probable benefit to the patient himself, for it is cruel to recommend what is beyond the resources of a patient, and oftentimes more sacrifice than should be is made to carry out ill-considered advice of the physician.

In reckoning the expense of change it must be considered: (1) whether it is to be temporary or permanent; (2) whether the patient can lead an idle life, or must engage at once in some remunerative occupation; or, having remained idle for a certain time, there would be a good prospect of his resuming some employment.

In answering the first question, it must be borne in mind that, as a rule, if an arrest of the disease is sought, a climate should be selected, if possible, in which the patient can remain throughout the year; if palliation only is sought, then a mild climate might be chosen in winter, which would be utterly unbearable in the summer.

It is not always wise to tell a patient on leaving home that his stay is to be indefinite, but when once he is away it is not difficult to prolong his stay; in fact, if the invalid improves he is very apt to become attached to the spot where the improvement occurs. In answering the second question, it should be remembered that while an absolutely idle and indolent life is necessary for some patients, at least during the time of serious constitutional disturbances, there are many patients free from such disturbances, who are better for occupation, provided it keeps them a good deal in the open air. In-door life diminishes enormously the chances of improvement from change of climate.

For many years the one idea of the practitioner was to get his pulmonary patients into a mild climate, one soothing to cough, and this notwithstanding the fact that consumption was rife among the natives of the land to which they were sent. In more recent years, it having been found that elevated regions show a remarkable exemption from the disease among their natives, it is becoming too much the custom to send all consumptives thither, without regard to their condition, ignoring the fact that the climatic conditions best suited to pulmonary diseases vary with the nature and condition of each case, and that those required for prophylaxis may be very different from those demanded for the treatment of advanced disease.

Patients with pulmonary tuberculosis present a great variety of conditions, of which the following are the principal types:

(1) Those presenting the earliest physical signs of tuberculosis of the apex, who have as yet shown little, if any, general disturbance from the disease, and who complain only of morning cough and expectoration. Threatened cases of hereditary disease, in which there are as yet no morbid physical signs, and not much constitutional disturbance, may usually be considered with this class.

(2) Patients with more advanced disease, showing some consolidation but no excavation, nor any serious constitutional disturbance.

(3) Hemorrhagic cases, that is, patients in whom pulmonary hemorrhage has been perhaps the earliest, and a frequently recurring symptom, but in whom there is as yet no marked febrile reaction, nor much physical evidence of disease.

(4) Patients with advanced disease, those with cavities or severe hectic symptoms.

(5) Patients in an acute condition.

(6) Cases of so-called fibroid or interstitial pneumonia.

(7) Patients recovering from acute pleurisy or pneumonia, in which the eruption of tubercle is dreaded.

(8) Patients in which the tubercular process has seriously invaded the larynx.

(9) Those with complications of other diseases, heart, renal, intestinal, rheumatic, etc.

The first object to be secured is an out-door life in a pure air. This undoubtedly has a beneficent local effect, but the chief good comes through the general improvement in nutrition produced by an open-air life, and this is much increased by the ability of the patient to take exercise, and it reaches its maximum benefit when he can lead an active, out-door life at considerable elevation (4,000 to 8,000 feet above the sea-level).

It will be seen, as we go over the different classes of cases with reference to their individual indications, that some of them are not suited to the severity of the mountain-air treatment. Changes of temperature in the mountains are often very great and very sudden, and though equability is not of so much importance as was formerly supposed, yet a certain amount of vigor is necessary to re-act properly to them. There seems little doubt that in suitable cases the improvement in nutritive activity is much more marked in mountainous regions than on the plains. There are probably several causes for this: (1) In a properly selected place, the air is rarified, causing increased respiratory activity. (2) The air is very dry and very pure. (3) The number of clear days when the invalid can be out-doors is vastly in excess of those on the plains.

Certain modifying influences which pertain to the individual have always to be borne in mind. It can be easily understood that a more rigorous climate, one involving the necessity of more active exercise, can be recommended to men than to women. The difficulty of securing sufficient out-door life for women, even in a mild climate, constitutes one of the chief factors in the relatively bad prognosis in tuberculosis in women as compared with men. Patients with much bronchial irritability often do less well in a very high altitude than in a lower one, and the same is sometimes true of a decidedly neurotic temperament. Age also must be considered in reference to possible removal to high altitudes. A very high elevation should not, as a rule, be recommended to a patient over fifty years of age.

Wherever the patient goes he should, if possible, consult some good physician of the region, who will lay out for him a plan of life. Many patients make themselves sick, and even destroy their chance of recovery, by neglecting to consult a local authority for this purpose.

We will now consider the indications furnished by the types of the disease as before classified.

(1) Those presenting the earliest physical signs of chronic tuberculosis of the apex, who have as yet shown little, if any, general disturbance from the disease, and who complain only of morning cough and expectoration.

It is this class of cases especially which shows the effect of improved ideas of treatment. The change from the old plan of enforced invalidism to an active out-door life has brought about many cases of arrest in this stage of the disease. It is, perhaps, not saying too much to say that the prognosis has been changed as regards this class of cases from very bad to very good.

While I have had such patients do well in different climates, some of them without leaving home, the results have averaged far better, in my experience, in those who have sought mountain climate than in those who have pursued any other course. The region which I have found best for this kind of treatment is the eastern slope of the Rocky Mountains, in the States of Colorado and New Mexico, where the altitude ranges from 4,000 feet to 8,000 feet.

The question will naturally be asked whether the patient should go at once from the sea-board to such a high elevation, or make a number of stops on his way out, in order to become accustomed to the diminished pressure. I have never known any ill effect in patients of this class from making the change at once; but it is especially necessary that they should consult a good local medical adviser at once, that they may be guided from the beginning, particularly in regard to the kind and amount of physical exercise which they should take.

(2) Patients with more advanced disease, showing some consolidation but no excavation, nor any serious constitutional disturbance.

The mountain climate is suited to many of this class also, and it is fortunate if they are in condition to try it, but if considerable area of one lung, or the apices of both are consolidated, and there is well-marked constitutional disturbance, if the pulse and temperature are both constantly above 100, then it may be well to try some low altitude first. For very low elevations, the dry, rather stimulating air of Aiken and its vicinity, or the pine regions of southern Georgia, may be recommended for the greater part of the year, the patient going north in the summer. When quiescence in the morbid process is established a move to the higher altitudes should be made.

(3) Hemorrhagic cases, that is, patients in whom a pulmonary hemorrhage has been perhaps the earliest and a frequently recurring symptom, but in whom there is as yet no marked febrile reaction nor much physical evidence of disease.

This class seems particularly suited to the high altitude treatment. Contrary to the old idea, these patients appear to be less liable to hæmoptysis in high altitudes than on the plains. I do not remember any patient of this class in whom the tendency seemed increased by the removal to a high altitude, and although such patients are usually advised to make several stops on their journey upward, I doubt if this precaution is often necessary. Of course it will be understood that what is said in this connection does not refer in any way to the hæmoptysis from rupture of a large vessel in a cavity of advanced disease.

(4) Cases of advanced disease, those with cavities or severe hectic symptoms.

Patients of this class had better, as a rule, stay at home; certainly if they are sick enough to be confined to the house. They can usually be made much more comfortable in their own homes than at any health resort, yet I have sometimes advised that such a patient with a very constant and harassing cough be sent to the moist climate of Florida, and the relief to the cough has more than compensated for the want of some home comforts. A poor patient, or one without abundant means even, should not be given such advice.

(5) Patients in an acute condition.

These may be quite different in their nature and requirements. We find (*a*) cases of acute general infiltration. These patients should be kept at home definitely. (*b*) Cases which begin violently with high fever and marked consolidation of lung, resembling pneumonia. Patients of this class should be kept at home till after the subsidence of the acute symptoms, and then may be removed to some low, dry place; afterward increasing elevations may be carefully tried. (*c*) Cases of acute exacerbation during the progress of chronic disease. Patients of this class should remain at home during the acute stage, going, perhaps, to some mild, sedative climate during its decline; but as soon as possible after the febrile disturbance is well over, if their condition otherwise warrants it, they should go to an elevated region.

(6) Cases of so-called fibroid or interstitial pneumonia. Special indications in these cases have to be considered. If the patient is young, and the heart is not enlarged, he may be sent to high elevations. If he is over fifty years of age, or if his heart is dilated, or if his cough is very harassing, a lower altitude should be chosen. Southern California offers excellent places for such, with varying elevation and moisture to suit individual symptoms.

(7) Patients recovering from acute pleurisy or pneumonia in whom the eruption of tubercle is feared. High elevations is the place *par excellence* for these. The increased respiratory and consequent increased nutritive activity are exactly what is wanted to prevent the development of chronic disease.

(8) Patients in whom the tubercular process has seriously invaded the larynx.

Such patients should be recommended mild, and even moist, climates, and on no account be sent to high altitudes. Southern California answers the purpose well. The dry air of high altitudes, however much good it may do by stimulating general nutrition, usually proves so great a local irritant to the larynx that incessant cough ensues, or, if the disease is situated high in the larynx, the swelling and ulceration of the cartilages are aggravated so that severe dysphagia and insufficient nourishment ensue.

(9) Those with complications of other diseases.

In regard to these a good deal of care has to be exercised oftentimes. In case of cardiac affection it may be said that while marked dilation should prevent a patient's being sent into a high altitude, it is not necessary to exclude every one from such who has a cardiac murmur, or who even is known to have organic valvular disease, with moderate hypertrophy, but such patients should be carefully watched and regulated in their habits, and should not be sent into the very highest altitudes.

In regard to renal disease, while it is admitted by the resident physician that *acute* nephritis is severe in high altitudes, they do not admit that patients with chronic disease are made worse, but claim rather that they are benefited by a residence there.

Patients with intestinal ulceration are said to do badly in high altitudes, but they do badly everywhere.

In regard to the rheumatic diathesis, it may be said that acute rheumatism is thought to be rather prevalent and severe in high altitudes, and such a tendency might turn the balance in favor of a lower resort. On the other hand, the chronic form of rheumatism does not seem to be made worse by elevation.

---

SOCIETY PRACTICE IN NEW ORLEANS.—The *New Orleans Medical and Surgical Journal*, June, 1888, says in an editorial: "The custom followed by so many physicians in this city of taking 'society practice' has grown to such an extent as to threaten destruction to all legitimate work by those few who value the dignity or well-being of the profession. Four-fifths of the people of this city are banded together into so-called 'Benevolent Associations,' whose objects are weekly indemnity while sick, free medical services, including drugs, and free burial in case of death. Every one of these societies annually chooses a physician, a druggist, and an undertaker, and always after fierce competition. Just before the annual election a committee goes around to some fifteen or twenty physicians, and the same number of druggists and undertakers, and requests each of these functionaries *to put in a bid.* Then begins the scramble. One physician will offer 'to do the work,' which includes attention to the families of the members, for four dollars per member per annum; another will bid three, another two, and so on until, as actually happened a few weeks ago, the final and successful bidder sees a profit and honor in the job at 'forty cents (40c.) per member per annum, payable quarterly.' "

---

Bill Nye declined an invitation to the Indian Medical Society by the following telegram: "Sorry cannot be there. May you and your associates continue to take life as easily as heretofore."

